# Post-infectious sequelae after Campylobacter enteric infection: a pilot study in Maricopa County, Arizona, USA

**DOI:** 10.1186/s40814-018-0335-z

**Published:** 2018-08-22

**Authors:** Erika Barrett, Dametreea Carr, Melanie L. Bell, Kristen Pogreba-Brown

**Affiliations:** Department of Epidemiology and Biostatistics, Mel and Enid Zuckerman College of Public Health, 1295 N Martin Ave, Tucson, AZ 85719 USA

**Keywords:** Campylobacter, Chronic sequelae, Pilot, Campylobacteriosis, Gastroenteritis

## Abstract

**Background:**

Campylobacter is a leading cause of gastroenteritis across the globe caused by the ingestion of contaminated food, water, or contact with animals carrying Campylobacter bacteria. The resulting disease, campylobacteriosis, is usually self-limiting, but cases may develop post-infectious sequelae (PIS) such as gastrointestinal disorders, neurological disorders, and joint disorders. The objective of this study was to estimate a crude incidence rate for PIS among Campylobacter cases in Maricopa County, Arizona, USA and to determine the feasibility of conducting a larger scale study to understand chronic outcomes from campylobacteriosis and salmonellosis.

**Methods:**

The pilot study spanned from August 1, 2016, to August 31, 2017. During this time, cases of campylobacteriosis were reported to the Maricopa County Department of Public Health and interviewed by public health students at the University of Arizona. Initial interviews were conducted using a routine enteric surveillance questionnaire, and eligible cases were recruited and consented into the pilot study. Follow-up with a questionnaire occurred 4 to 6 weeks from the date of each case’s initial interview. Data analysis was conducted using STATA SE 14 and included chi-squared tests to determine differences in demographics, symptoms, and exposures between those enrolled in the study and those eligible but not enrolled during the study period and feasibility metrics for the study including enrollment rates, response rates, time to interview, and reasons for non-enrollment. Crude rates with 95% confidence intervals were calculated to estimate PIS.

**Results:**

Of the routine surveillance cases, 102 (36%) enrolled into the pilot study. Of enrolled participants, 68.6% completed the follow-up questionnaire. Most enrolled participants were non-Hispanic White, male, and aged 60 + years. Over half (52.8%, 95% CI 41.1%, 64.5%) of cases experienced PIS approximately 4 to 6 weeks after acute onset of campylobacteriosis.

**Conclusions:**

Results from this pilot study indicate that a larger study is feasible. The larger study will identify the true incidence of PIS and improve the management of patient health among ethnically diverse populations.

**Electronic supplementary material:**

The online version of this article (10.1186/s40814-018-0335-z) contains supplementary material, which is available to authorized users.

## Background

Campylobacter is the leading cause of bacterial gastroenteritis in the world and is transmitted to humans through contaminated food, water, or exposure to animals who may be carrying the bacteria [1]. The disease is generally self-limiting with cases experiencing symptoms 1 week or less, followed by a full recovery. However, for a minority of people, these symptoms can extend for months and even years or can develop into post-infectious sequelae (PIS) such as Guillan-Barré syndrome, reactive arthritis, and irritable bowel syndrome [2–4].

There are very few prospective studies in the U.S.A. that examine the incidence rate of these outcomes among cases of Campylobacter and none in ethnically diverse populations [2, 5, 6]. Prospective studies are necessary to establish cause and effect due to a variety of factors related to these infections and subsequent PIS. First, neither of these pathogens produce long-lasting antibodies that can reliably be used as a marker for previous infection [7]. Next, there have been some studies that have linked the severity of acute symptoms, such as hospitalization, the number of days with diarrhea, and occurrence of bloody diarrhea to these chronic outcomes [4, 8]. Collecting acute information at the time of the event greatly decreases recall bias and response variability of acute symptoms [9]. Many PIS do not develop for weeks to even months after the initial infection [2, 4] further adding to the challenge of recall bias related to symptoms and pertinent exposures. In addition, many of these diagnoses have other known associations, such as depression, and are based on a set of symptomatic criteria (e.g., Rome IV for irritable bowel syndrome [10]) and not a diagnostic test that would link them directly to a previous infection. Prospective studies allow for the identification of exposure (infection), and tracking the onset, duration, and recovery time of these outcomes accurately. To understand the rate of chronic outcomes following a Campylobacter infection, we conducted a 1-year pilot study of campylobacteriosis cases in Maricopa County, Arizona, USA in preparation for a larger study. This larger study will take advantage of prospective collection of epidemiologic (through questionnaires) and microbial data (through follow-up stool cultures) and will fill several knowledge gaps regarding specific microbial factors that translate into clinical outcomes. Patients with Salmonella and Campylobacter infections in Arizona and Colorado will be recruited for 15 months including two summers to increase enrollment.

The aim of this prospective cross-sectional pilot study was to determine a crude incidence rate of PIS (e.g., functional gastrointestinal disorders, neurological outcomes, and joint disorders) among campylobacteriosis cases and determine the feasibility of conducting a follow-up questionnaire in this population. The objectives of this study are to (1) determine the feasibility of conducting follow-up surveys in the study population to determine recruitment rate, response rate, and acceptability for the larger study, (2) identify any associations between demographics and other risk factors for those who enrolled in the study versus those who did not, and (3) estimate the rate of chronic outcomes in the study population.

## Methods

Following a patient’s clinical visit where a stool sample is first collected, laboratories report all confirmed and probable cases of campylobacteriosis to the local health department in accordance with state law. This notification usually occurs within 1–2 days of testing and 3–4 days of the initial patient visit. As part of an ongoing routine surveillance contract with the Maricopa County Department of Public Health (MCDPH), all cases of campylobacteriosis are assigned to the Student Aid for Field Epidemiology Response (SAFER) for follow-up. This program embedded in the university’s College of Public Health, teaches students about outbreak response and public health surveillance [11]. Each case was then interviewed over the telephone utilizing a routine public health enteric surveillance questionnaire (EQ) that collects information on demographics, acute symptoms, pre-existing conditions, and exposures to food, water, and animals [12]. Following the EQ, cases were recruited and consented into the pilot study from August 1, 2016–August 31, 2017 in order to collect 13 months of data across two summers when case counts are normally high (see Additional file 1). To be eligible for the study, participants must have been 18 years of age or older, speak English or Spanish, and a confirmed or probable Campylobacter case from Maricopa County, Arizona with a completed EQ [12]. Participants were not compensated for their participation. The University of Arizona Institutional Review Board (IRB) reviewed and accepted this research project with a determination of exempt under 45 Code of Federal Regulations (CFR) 46.101(b) [13].

The participant’s EQ unique identifier was also used in the cohort questionnaire (CQ). The CQ was projected to take 5–10 min of time and included 8–22 questions depending on number of reported symptoms at the time of the interview (see Additional file 1). Two final questions in the CQ were used to understand feasibility of follow-up stool samples and acceptability of alternative stool collection methods. No follow-up stool samples were collected for the pilot. Cases who agreed to participate were entered into a separate call log and contacted starting 4 weeks from EQ or onset date, whichever was sooner. Attempts were made to contact participants up to five times at different dates and times, with at least one evening or weekend call, before they were indicated as lost-to-follow-up. All interviews (EQ and CQ) were conducted orally over the phone in English or Spanish utilizing an online version of the questionnaire in the survey software Qualtrics [14], with trained staff and students in a secure call center.

After the CQ for all enrolled participants was complete, data from the CQ was linked to the de-identified EQ using the unique identifier common in both datasets. Demographic data and rates of PIS (gastrointestinal symptoms, joint symptoms, neurological symptoms, and other symptoms) were determined for the eligible and enrolled groups. Chi-squared tests were used to determine differences in demographics, symptoms, and exposures between those enrolled in the study and those eligible but not enrolled during the study period. Feasibility metrics for the study including enrollment rates, response rates, time to interview, reasons for non-enrollment, and CQ information were calculated. All analyses were conducted using STATA SE 14 (College Station, TX) [15].

## Results

**Fig. 1:**
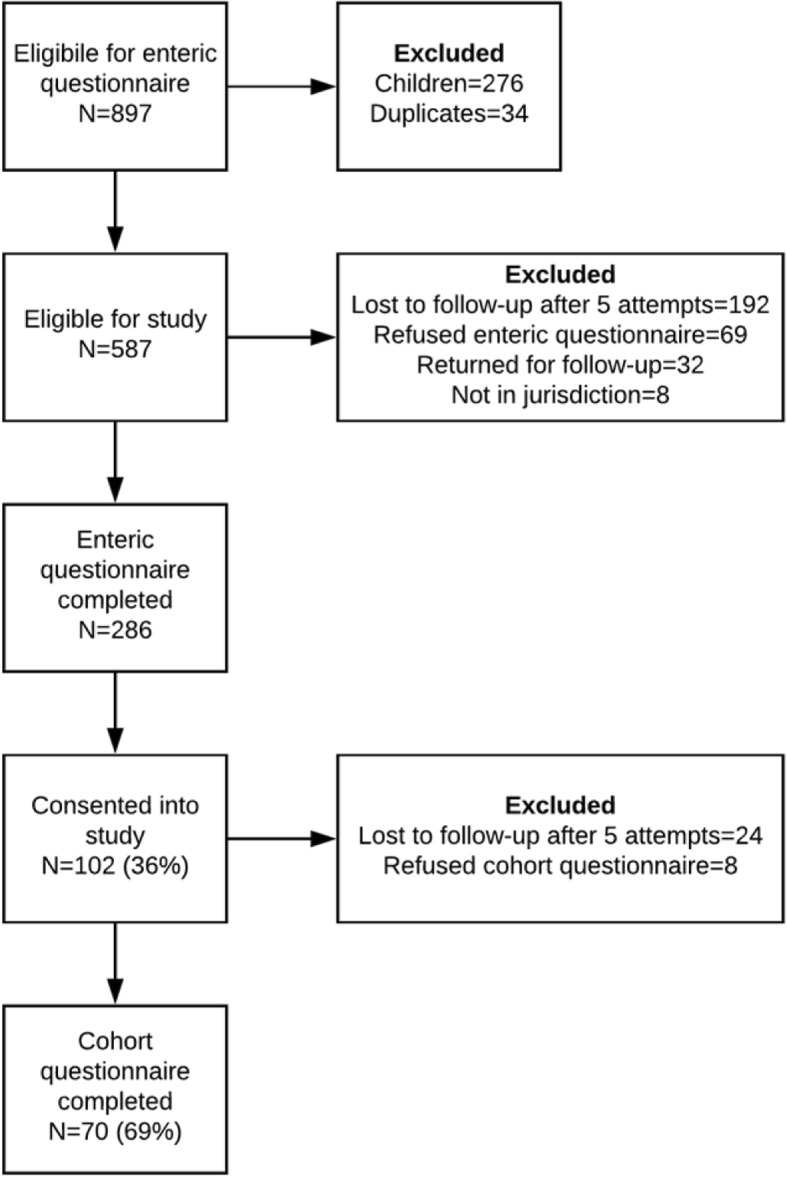
Flowchart of participation in study

Of the 587 eligible patients, 286 (48.7%) were interviewed by SAFER students using the EQ, and 102 (36%) were enrolled in the study from August 1, 2016–August 31, 2017 (Fig. 1). Key reasons for non-enrollment included cases who were lost to follow-up (40.3%), cases who were not interested in participating (20.9%), cases who refused to take the EQ (17.1%), and cases who were not asked to participate in the study (9.2%). Of the twenty-eight cases who were not asked to participate in the study, reasons for not being asked to participate included cases being limited on time and rushing off the phone at the end of the EQ (14.3%), cases ending the phone call after only partial completion of the EQ (7.1%), student surveyors neglecting to ask if cases were interested in participating before ending the call (67.9%), lack of sufficient training on the enrollment protocol (3.6%), and other undocumented reasons (7.1%). Twenty-two cases (7.2%) had unknown reasons for not enrolling in the follow-up study. Seventy participants (69%) enrolled in the study and completed the CQ from August 15, 2016–October 4, 2017. On average, participants were contacted 28 days from the EQ (range 13–55 days) and 57 days from their onset date (range 32–168 days). The CQ took 12 minutes on average (range 1–34 minutes). Of the eight participants who refused the CQ, the primary reason was due to confusion: cases thought they had already taken the survey and did not want to take it again or did not understand the purpose of the survey. Almost half of the enrolled participants completed the CQ on the first call attempt (36%) or the second attempt (23%). Five (7%) of the CQ were completed in Spanish. After five call attempts during the evenings and weekends, 24 participants were lost to follow-up.

No significant differences in demographics between those enrolled and those eligible but not enrolled were found (Table 1). Most were in the ≥ 60 years of age category (33.2% eligible, and 38.2% enrolled). Sex differed between those eligible and those enrolled (43.5% male for eligible and 53.9% male for enrolled). Most identified as non-Hispanic (69.6% eligible and 76.5% enrolled). Most cases described their community as urban or suburban (84.2% eligible and 83.4% enrolled).

**Table 1:** Demographics between cohorts

Characteristic	Eligible (not enrolled) *N* = 184 *N* (%)	Enrolled *N* = 102 *N* (%)	*p* value*
Age			0.83
18–29	37 (20.1)	16 (15.7)	
30–39	28 (15.2)	14 (13.7)	
40–49	29 (15.8)	15 (14.7)	
50–59	29 (15.8)	18 (17.7)	
≥ 60	61 (33.2)	39 (38.2)	
Gender			0.09
Male	80 (43.5)	55 (53.9)	
Female	104 (56.5)	47 (46.1)	
Race			0.40
White	150 (81.5)	82 (80.4)	
African American	5 (2.7)	1 (1.0)	
Asian	7 (3.8)	2 (2.0)	
Native Hawaiian/Pacific Islander	0 (0)	0 (0)	
American Indian	5 (2.7)	1 (1.0)	
Other	12 (6.5)	13 (12.8)	
Unknown	1 (0.5)	0 (0)	
Ethnicity			0.35
Non-Hispanic	128 (69.6)	78 (76.5)	
Hispanic	48 (26.1)	20 (19.6)	
Unknown	8 (4.3)	4 (3.9)	
Location of Residence			0.32
Urban	81 (44.0)	43 (42.2)	
Suburban	74 (40.2)	42 (41.2)	
Town	14 (7.6)	9 (8.8)	
Rural, not a farm	10 (5.4)	3 (2.9)	
Farm	1 (0.6)	4 (3.9)	
Unknown	4 (2.2)	1 (1.0)	

*Chi-squared test comparing those eligible but not enrolled and enrolled

Fifty-five (79.1%) participants agreed that they would be willing to provide a follow-up stool sample if provided all collection materials and a $40 gift card incentive. Additionally, 35 participants (52.2%) said they would be more likely to participate in the follow-up stool sample if they were able to provide their sample via a wipe rather than the traditional stool collection method, and 22 (32.8%) noted that they would not be willing to participate and the method available would not affect their willingness to participate.

Differences among symptoms and previous medical conditions between those who enrolled in the study and those eligible but not enrolled are outlined in Table 2. Those who reported mucousy diarrhea (*X*^2^ (1, *N* = 87) = 4.54, *p* = 0.03), fever (*X*^2^ (1, *N* = 68) = 3.86, *p* = 0.05), and chills (*X*^2^ (1, *N* = 21) = 14.7, *p* = 0.0001) were significantly more likely to enroll into the study. Additionally, those with no chronic conditions (*X*^2^ (1, *N* = 41) = 4.86, *p* = 0.03) and other chronic conditions ((*X*^2^ (1, *N* = 53) = 6.51, *p* = 0.01) were significantly more likely to enroll in the study. When investigating other chronic underlying conditions, enrolled participants reported thyroid (5.8%) and hypertension (5.8%) problems more than those not enrolled (0.6% and 0.6% respectively). No other significant differences in symptoms or previous medical conditions were found.

**Table 2:** Symptoms and exposure between cohorts

Symptoms and exposures	Eligible (not enrolled) *N* = 184 *N* (%)	Enrolled *N* = 102 *N* (%)	*p* value*
Diarrhea	177 (97.8)	100 (98.0)	0.89
Bloody diarrhea	45 (24.5)	28 (27.5)	0.58
Watery diarrhea	66 (35.9)	34 (33.3)	0.67
Mucousy diarrhea	137 (74.5)	87 (85.3)	**0.03**
Fever	102 (61.8)	68 (73.9)	**0.05**
Nausea	102 (57.6)	62 (63.3)	0.36
Vomit	70 (38.9)	41 (41.4)	0.68
Abdominal pain	147 (80.33)	89 (89.0)	0.06
Headache	104 (57.5)	64 (64.7)	0.24
Other symptoms	79 (42.9)	62 (60.8)	**0.004**
Chills	20 (7.0)	21 (20.6)	**0.0001**
Antibiotics in month prior	24 (13.6)	8 (7.8)	0.15
Antacids in month prior	70 (39.6)	38 (38.0)	0.80
Healthcare outcomes			0.68
Primary care	79 (43.2)	49 (48.5)	
Hospitalization	60 (32.8)	28 (27.7)	
ED/urgent care only	43 (23.5)	24 (23.8)	
Chronic condition			
None	99 (53.8)	41 (40.2)	**0.03**
IBS	8 (4.4)	2 (2.0)	0.29
Chronic gastritis	1 (0.5)	1 (1.0)	0.67
Diabetes	20 (10.9)	13 (12.8)	0.63
Arthritis	6 (3.3)	7 (6.9)	0.16
Other	67 (36.4)	53 (51.9)	**0.01**
Thyroid	2 (0.6)	6 (5.8)	**0.02**
Hypertension	2 (0.6)	6 (5.8)	**0.02**

*Chi-squared test comparing those eligible but not enrolled and enrolled, bolded variables indicate statistically significant differences between groups

In total, 37 (52.8%, 95% CI 41.1, 64.5) patients had some form of post-infectious sequelae approximately 6 weeks after their acute onset of disease (Table 3). Symptoms from their acute infection were ongoing for seven participants at the time of the CQ; this was down from 21 participants who had ongoing symptoms when first interviewed approximately 1 week after symptom onset. At time of the CQ, 24 patients (34.3%, 95% CI 23.2, 45.4) sought additional medical care, and 16 of those (66.6%) were provided a diagnosis (e.g., Crohn’s disease, gastric cirrhosis, colitis, fibromyalgia, irritable bowel syndrome). Seventeen cases (24.3%, 95% CI 14.3, 34.3) had new or continuing GI symptoms, 9 (12.8%, 95% CI 5.0, 20.6) had new or continuing joint symptoms, 4 (5.7%, 95% CI 0.3, 11.1) had new or continuing neurological symptoms, and 7 (10.0%, 95% CI 3.0, 17.0) had other symptoms (i.e., headaches, swelling, fatigue, etc.).

**Table 3:** Proportion of post infectious sequelae in enrolled group (*N* = 70) and 95% confidence intervals for the proportions

Post infectious sequelae (new or continuing) *	*N* (%)	95% Confidence intervals
All	37 (52.8)	41.1, 64.5
Sought additional medical care	24 (34.3)	23.2, 45.4
Gastrointestinal symptoms	17 (24.3)	14.3, 34.3
Joint symptoms	9 (12.8)	5.0, 20.6
Neurological symptoms	4 (5.7)	0.3, 11.1
Other symptoms	7 (10.0)	3.0, 17.0

*See Additional file 1 for questions used

## Discussion

The objective of this pilot study was to determine the rate of chronic outcomes resulting from Campylobacter infection and assess feasibility for a larger study by evaluating recruitment and retention rates. We found that 52.8% of patients experienced PIS 6 weeks after acute onset of disease, with seven participants experiencing ongoing symptoms at the time of the CQ. After assessing recruitment and retention rates, we found that 36% of eligible patients enrolled into the study without incentives and 68.6% of those enrolled completed the CQ. Overall, the pilot study results indicate that a larger, definitive study is feasible.

Regarding any differences between those who enrolled and those who did not, the authors can only make an educated guess given our long history of interviewing patients. We would assume that those who had additional symptoms would be more inclined to enroll because they feel it is a more serious condition. However, higher rates of enrollment among those without pre-existing conditions may be because this is a rare event for them and holds some interest. Both observations could also be an artifact of a small sample size and not hold true in a larger study. Interestingly, these two risk factors, severity of disease and history of other chronic conditions have been noted in the literature to increase the odds of developing a PIS so while one would bias the results towards finding an association, the other would bias them away from this. To address these issues, we have developed a more robust recruitment and retention plan for the larger study as described below.

We estimated that 10–15% would experience PIS following acute onset of disease; however, our pilot study found that 52.8% of patients experienced PIS 6 weeks after acute onset. This is a much larger proportion compared to other literature [2, 16]; however, this pilot study used only self-reported symptoms and not diagnosed conditions which would lead to higher numbers. There are few prospective studies investigating PIS after Campylobacter infection; estimated rates for reactive arthritis after Campylobacter infection may occur in 1–5% of cases, 1–11% for irritable bowel syndrome, and less than 1% of cases for Guillain Barré syndrome [2]. The larger study will contribute significantly to this field by providing estimates of PIS utilizing a prospective study design. The larger study will also include Salmonella cases, a similar bacterial infection also linked to PIS. Salmonella cases were not used for this pilot study because at the time their assignment to SAFER was different from Campylobacter cases, and we wanted to simplify the protocol for testing. In addition, our larger study will include a clinician who can determine if the reported symptoms would lead to a clinical diagnosis for IBS or other commonly reported PIS.

Methods of follow-up for prospective cohort studies are a major challenge to ensure validity of study results. To achieve our recruitment rate goal of 65%, the larger study will provide a graduated incentive system for participants, text reminders, e-mail or mail updates on the study, a study web portal, and the ability for participants to report symptoms or diagnoses in real-time through a secure online questionnaire. Information on engagement as well as outputs from the online questionnaire will be shared with interviewers before the next scheduled CQ to help with retention. To reduce the number of participants who are lost to follow-up, we will use “assigned” interviewers so that participants are more familiar with one key team member, like a caseworker. Given the largest group enrolled in the study was in the 60 + age group, this will help reduce confusion and provide consistency across multiple interviews.

In our larger study, we plan to collect follow-up stool samples to identify biomarkers for chronic outcomes and understand how the gut microbiome has changed after acute infection. Regarding feasibility of these follow-up stool samples, cohort participants stated that they would be willing to provide us with a follow-up stool culture if provided a $40 incentive. Additionally, more people will be willing to participate if they are provided the option to use a wipe instead of traditional collection methods. We plan to provide these options in the larger study to help with retention and sample collection. Additionally, since follow-up stool samples will occur at home, we anticipate high acceptability and compliance in comparison to other prospective cohorts [17].

This pilot study has strengths and limitations. The prospective design of this pilot study allowed for the tracking of PIS after acute infection. Additionally, the student interviewers who participated in the study gained valuable experience in public health research. Due to the small sample size, extrapolations on the extent and severity of post-infectious sequelae are not recommended. Over half of the cohort had PIS at time of CQ; this is much larger than was expected and is in contrast to other studies [4, 18–22] that have examined similar outcomes. We attribute this higher proportion to a few things. First, the questions we selected were intended to capture a broad range of symptoms for gastrointestinal, neurological, and joint symptoms; the larger study will utilize validated questionnaires for PIS. Also, PIS, such as irritable bowel syndrome, can fluctuate over time. We only conducted one CQ with patients at 4–6 weeks post-acute infection, therefore the high proportion of PIS in our cohort may be an artifact of the acute infection including ongoing symptoms. The future study will conduct multiple CQs with patients over the course of a year to account for variability in PIS. Finally, those who enrolled in the study were healthier with fewer chronic conditions and had mucousy diarrhea, fever, and chills more often than those not enrolled. Participants enrolled were educated on the possibility of long-term symptoms at the time of enrollment, and therefore may have been more aware of changes or ongoing symptoms at the time of CQ.

## Conclusions

This pilot study will be used to address the larger issue of determining the true burden of disease following infection with common foodborne pathogens. Generally, Campylobacter and Salmonella are considered self-limiting acute infections, and chronic outcomes are largely ignored from a clinical or public health standpoint. Our study will be used to determine the true incidence of these long-term sequelae in an ethnically diverse population in Arizona and Colorado improving the generalizability of the results. Finally, we will team with microbiologists to determine the specific mechanisms that Campylobacter and Salmonella use to initiate autoimmune or other chronic outcomes. Improved understanding of these concepts will lead to improved management of patient health.

## Additional file

Additional file 1: Protocol, Recruitment Script, and CQ. (DOCX 27 kb)

## Abbreviations

CQ: Cohort questionnaire
EQ: Enteric surveillance questionnaire
MCDPH: Maricopa County Department of Public Health
PIS: Post-infectious sequelae
SAFER: Student Aid for Field Epidemiology Response

## References

[1] Acheson D, Allos BM (2001). Campylobacter jejuni infections: update on emerging issues and trends. Clin Infect Dis.

[2] Keithlin J, Sargeant J, Thomas MK, Fazil A (2014). Systematic review and meta-analysis of the proportion of Campylobacter cases that develop chronic sequelae. BMC Public Health.

[3] Batz MB, Henke E, Kowalcyk B (2013). Long-term consequences of foodborne infections. Infect Dis Clin N Am.

[4] Riddle MS, Gutierrez RL, Verdu EF, Porter CK (2012). The chronic gastrointestinal consequences associated with Campylobacter. Curr Gastroenterol Rep.

[5] Townes JM, Deodhar AA, Laine ES, Smith K, Krug HE, Barkhuizen A (2008). Reactive arthritis following culture-confirmed infections with bacterial enteric pathogens in Minnesota and Oregon: a population-based study. Ann Rheum Dis.

[6] Spence MJ, Moss-Morris R (2007). The cognitive behavioural model of irritable bowel syndrome: a prospective investigation of patients with gastroenteritis. Gut.

[7] Constantiniu S, Romaniuc A, Chiriac R, Berea C, Kalis O, Rezus E (2008). Antibacterial antibodies for some enterobacteria in sera of patients with reactive arthritis and other rheumatoid diseases. Roum Arch Microbiol Immunol.

[8] Gradel KO, Schonheyder HC, Lundbye-Christensen S, Ejlertsen T, Nielsen H (2009). Severity of human non-typhoid salmonellosis as a predictor of short- and long-term mortality. Scand J Infect Dis.

[9] Garg AX, Marshall J, Salvadori M, Thiessen-Philbrook HR, Macnab J, Suri RS (2006). A gradient of acute gastroenteritis was characterized, to assess risk of long-term health sequelae after drinking bacterial-contaminated water. J Clin Epidemiol.

[10] Drossman DA (2006). The functional gastrointestinal disorders and the Rome III process. Gastroenterology.

[11] Pogreba-Brown K, Harris RB, Stewart J, Anderson S, Erhart LM, England B (2010). Outbreak investigation partnerships: utilizing a student response team in public health responses. Public Health Rep.

[12] Arizona (2008). Arizona Administrative Code A.R.S. § 32-501 et seq. 4.

[13] Health and Human Services (2009). 45 CFR 46.101(b).

[14] Qualtrics (2014). Provo, Utah: Qualtrics Research Suite.

[15] StataCorp (2015). Stata Statistical Software: Release 14.

[16] Porter CK, Choi D, Cash B, Pimentel M, Murray J, May L (2013). Pathogen-specific risk of chronic gastrointestinal disorders following bacterial causes of foodborne illness. BMC Gastroenterol.

[17] Keithlin J, Sargeant JM, Thomas MK, Fazil A (2015). Systematic review and meta-analysis of the proportion of non-typhoidal Salmonella cases that develop chronic sequelae. Epidemiol Infect.

[18] Schultze A, Akmatov MK, Andrzejak M, Karras N, Kemmling Y, Maulhardt A (2014). Comparison of stool collection on site versus at home in a population-based study: feasibility and participants’ preference in pretest 2 of the German National Cohort. Bundesgesundheitsblatt Gesundheitsforschung Gesundheitsschutz.

[19] Pope JE, Krizova A, Garg AX, Thiessen-Philbrook H, Ouimet JM (2007). Campylobacter reactive arthritis: a systematic review. Semin Arthritis Rheum.

[20] Hannu T, Mattila L, Rautelin H, Pelkonen P, Lahdenne P, Siitonen A (2002). Campylobacter-triggered reactive arthritis: a population-based study. Rheumatology.

[21] Keithlin J, Sargeant JM, Thomas MK, Fazil AA (2015). Systematic review and meta-analysis of the proportion of non-typhoidal Salmonella cases that develop chronic sequelae. Epidemiol Infect.

[22] Porter CK, Thura N, Riddle MS (2013). Quantifying the incidence and burden of postinfectious enteric sequelae. Mil Med.

