# Supplementary material for: Post-infectious sequelae after Campylobacter enteric infection: a pilot study in Maricopa County, Arizona, USA
Source: Pilot Feasibility Stud. 2018 Aug 22;4:142. doi: 10.1186/s40814-018-0335-z (PMC6103860; doi:10.1186/s40814-018-0335-z)
Supplement: Supplementary file 1 — Protocol, Recruitment Script, and CQ. (DOCX 27 kb) [file 40814_2018_335_MOESM1_ESM.docx]

**Post-infectious sequelae after *Campylobacter* enteric infection: a pilot study in Maricopa County, Arizona, USA Project Protocol**

1. Check the Campylobacter case call log to see if an individual is eligible
   1. They must be over 18 to participate.
   2. They must be able to interview in English or Spanish.
2. If they are eligible, conduct the regular SAFER interview (provided as supplementary material) with the participant
3. At the end of the SAFER interview, complete the recruitment script (provided at end of protocol) with the participant
   1. If they agree to participate, try to conduct the **consent form over the phone**
      1. Go to “Consent Form” on the bookmarks bar.
      2. Explain the study and their involvement
      3. At the end of the survey, ask “Do you give me permission to type your name electronically as confirmation to participate voluntarily in this study?”
         1. If yes, sign the participants name, date, and click submit.
         2. If no, reply “To participate in this study you must complete the consent form. Can I send this to you electronically or through the mail?” then follow the steps in the recruitment script.
      4. At the end, tell them you will be sending them a copy of the consent form. Ask if they would prefer this electronically or through mail.
         1. If electronic, go to Qualtrics, click on Distributions, click on Emails.
         2. Click on the “Load Message” box, click on “Organization Library: University of Arizona “Campy FU Consent **Form**” (provided at end of protocol)
         3. Enter in their name and the date we will start calling them.
         4. Enter in their email address in a new panel.
         5. Label the Email “University of Arizona SAFER Consent Form”
         6. Save as the MEDSIS ID.
         7. Click send.
   2. If they agree to participate, but would like to **complete the consent electronically**
      - 1. Go to Qualtrics, click on Distributions, click on Emails.
        2. Click on the “Load Message” box, click on “Organization Library: University of Arizona “Campy FU Consent **E-mail**”
        3. Enter in their name and the date we will start calling them.
        4. Enter in their email address in a new panel.
        5. Label the Email “University of Arizona SAFER Consent Form”
        6. Save as the MEDSIS ID.
        7. Click send.
   3. If they agree to participate, but would like to **complete the consent through mail**
      - 1. Create a hardcopy packet for them which includes the Consent Form and a pre-paid envelope.
        2. Address the envelope to the participant.
4. After enrollment, copy and paste the participants line from the campy log, into Sheet 3 (PILOT Study). Complete Columns M, R, (if different than MEDSIS originally, W (if applicable), X and Y.
   1. X - Interview Date: this should be the day you complete the SAFER interview.
   2. Y - Date to start calls: Enter in the date that is 30 days from onset date. If it is beyond 4-6 weeks since the onset date, calculate 14 days from the interview date and use this date.

**Completing the Survey**

1. When you come into the call center, check the PILOT Study log to see if there are any calls to make.
2. If there is a call to be made, prioritize giving this individual a call.
3. Confirm that consent has been completed before calling.
4. Open the survey by clicking on “Campy Pilot Survey” on the bookmarks bar (provided as supplementary material)
5. Enter in the password
6. Complete the first set of questions before you call the person utilizing the information from the call log.
7. Contact the case utilizing the introduction script on the survey.
8. Complete the survey with the participant.

**Recruitment Script**

Thank you for taking the time to answer all of our questions. At this point in time, the health department has collected all the information we need. However, we would like to know if you would be willing to be contacted by investigators at The University of Arizona in four to six weeks? University researchers are conducting a short study to help determine what, if any, long term health outcomes you may experience following your recent infection. Would you be willing to answer a short follow-up survey if we/they called you back in about 4-6 weeks? This survey should take 5-10 minutes of your time and is completely voluntary.

*If No;* Thank you again for your time and have a nice day.

*If Yes;* Thank you for being willing to participate in this follow-up study.

Is it okay for us to share your name, phone number, and address with the researchers? This information will not be shared with anyone else and will be kept confidential.

*If No:* Okay. Thank you again for your time and have a nice day.

*If Yes:* There is a consent form that is required by The University of Arizona that outlines the study and your voluntary involvement. We would like to complete this with you ahead of time so that when we call back, we can complete the interview in even less time. Is it okay to complete this with you at this time?

*If No:* Can I e-mail you the link to complete the consent at a later time?

Enter in the e-mail into the spreadsheet and send them the consent link.

*If yes:* Okay! (Explain the purpose of the study and their involvement)

Do you give me permission to type your name electronically as confirmation to participate voluntarily in this study?

*If no*: To participate in this study you must complete the consent form. Can I send this to you electronically or through the mail?

***If yes*:** Great! Thank you for your time. I would like to send you a copy of the consent form for your records. Would you like this sent to your email or through mail?

Send them the consent form to their provided e-mail or address

Thank you! We will be contacting you in a few weeks to complete the survey with you. Thank you again for your participation in this study and have a great day.

*If they prefer to complete it electronically at a later time*: Okay, can I e-mail you the link to complete the consent at a later time?

*IF yes:* What is your current e-mail address?

We will send you the consent form. It should only take a few minutes of your time. Please complete the form by signing your name at the end and clicking submit.

*If they prefer a hard copy*: Okay, can I confirm your street address with you? When you receive the consent form, please send it back signed in the provided pre-paid envelope as soon as possible. If we have not received the consent form by the time we contact you, we will complete the consent with you on the phone prior to your participation in the study

**Frequently Asked Questions & Answers**

*How long will the follow-up interview take?*
Approximately 10 minutes

*What are the health outcomes I might experience?*
The vast majority of people who become infected with *Campylobacter* recover within a week without any long-term consequences. However, a small number of people experience longer lasting symptoms such as persistent diarrhea, joint pain and in even more rare cases, neurological symptoms. There is little known about the number of people in Arizona who experience these outcomes and we are trying to learn more about a person’s long-term health after these types of infections.

*What kind of questions will you be asking?*
The short follow-up survey will ask about any symptoms you may still have or have developed since today. We will also be asking about the general health of other people within your household.

*Who will have access to my personal information (or something related to this type of concern)?*No one except the research group at The University of Arizona will have access to your information.

*What are you going to do with the information I give you?*This project is a pilot study. A pilot study is a short study where researchers decide whether they can do a larger study in the future. Sometimes the results from a pilot study are published and sometimes they are used only to show that it is important to conduct a larger, more involved study. Your personal information will be completely confidential and will not be linked to your responses. However, your answers to the survey will help inform future research. Your involvement will help us design future research studies to help improve health outcomes for others.

*My doctor didn’t say anything about long term health outcomes/consequences. Do you have more information about this?*Your doctor likely did not mention any long term consequences because they are very rare. Yes, I have a website you can look at to learn more? (We can either direct them to CDC or another reputable site.) For the larger study we will create a study website for this with more information and links

**Qualtrics E-mail for Consent Forms**

Hello Name Here,

Thank you for agreeing to participate in the follow-up pilot study through the University of Arizona SAFER Team! In order for us to complete the survey with you, you must complete the short consent form below. This will only take a few minutes of your time. Please complete the form by typing in your full name and clicking submit.

**Follow this link to the Consent Form:**
${l://SurveyLink?d=Take the survey}

Or copy and paste the URL below into your internet browser:
${l://SurveyURL}
We will be contacting you the week of *MM/DD/YYYY* to complete the 5-10 minute survey.  Thank you for your participation!
Thank you again!
The University of Arizona SAFER Team

Follow the link to opt out of future emails:
${l://OptOutLink?d=Click here to unsubscribe}

Enteric Questionnaire (EQ) - Campy Follow Up

Start of Block: Default Question Block

Q35 **Complete the first set of questions before you call the person.  It will make the interview easier and faster.**

medsis MEDSIS ID (required)

________________________________________________________________

int_name Interviewer Name (Last, First)

________________________________________________________________

|  |
| --- |

int_date Date of Interview (MM/DD/YYYY)

________________________________________________________________

onset_date Onset Date (*from original interview*)

________________________________________________________________

Q34 Number of days from onset date to TODAY (it will be helpful to figure this out now)

________________________________________________________________

| 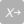 | 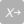 |
| --- | --- |

symptoms FROM ACUTE INTERVIEW - Had the primary symptoms resolved at the time of the interview?

- Yes, resolved after ___ Days (1) ________________________________________________
- No, Ongoing (0)

Q36 CONTACT CASE

Hello, I'm calling with the University of Arizona SAFER Team. A few weeks ago you had agreed to participate in a short follow-up survey that will ask you some questions regarding your recent Campylobacter infection. Do you still agree to participate at this time?

If yes,
Thank you!  We are interested in learning about the state of your health following your *Campylobacter* infection.

Display This Question:

If symptoms = 0

ongoing When we first spoke to you, you were still having ongoing symptoms,  Approximately how long did you continue to have gastrointestinal symptoms from the time you first got sick on ${onset_date/ChoiceTextEntryValue}?

- Days (1) ________________________________________________
- Still Ongoing (2)

text1 I am going to go through a list of general types of symptoms and if you started to develop any of these issues following your initial symptoms please let me know.  Ideally, we would like to know about when they started, how severe they have been and if they have stopped.  We are most interested in conditions related to any digestive problems, joint pain or any other neurological problems.

| 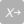 | 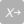 |
| --- | --- |

medcare First, since you were diagnosed with a *Campylobacter* infection, have you sought medical care for any additional reason?

- Yes (1)
- No (0)
- Don't Know (9)
- Prefer Not to Answer (99)

Display This Question:

If medcare = 1

| 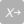 | 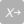 |
| --- | --- |

meddiag Did a healthcare provider give you a diagnosis?

- Yes (1)
- No (0)
- Don't Know (9)
- Prefer Not to Answer (99)

Display This Question:

If meddiag = 1

| 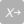 | 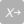 |
| --- | --- |

specdiag Were you diagnosed with any of the following?

- Irritable Bowel Disease (IBD) (1)
- Irritable Bowel Syndrome (IBS) (2)
- Guillan Barre Syndrome (GBS) (3)
- Reactive Arthritis (aka Reiter Syndrome) (4)
- Hemolytic Uremic Syndrome (HUS) (5)
- Auto-immune thyroid disease (6)
- Other (7) ________________________________________________

text2 Even if you have not been diagnosed with anything, we are still interested in the types of symptoms you may be experiencing.

| 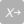 | 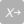 |
| --- | --- |

gisymp Since your initial symptoms stopped (and they may not have), have you had any continuous or newly developed **gastrointestinal or digestive problems**?

- Yes (1)
- No (0)
- Don't Know (9)
- Prefer Not to Answer (99)

Display This Question:

If gisymp = 1

ginat Can you describe the nature of your problems?

________________________________________________________________

Display This Question:

If gisymp = 1

| 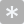 |
| --- |

gidur How long did the symptoms last? (Days - if symptoms ongoing, determine the number of days from onset to interview date - *only put number in box*)

________________________________________________________________

Display This Question:

If gisymp = 1

gicurr Did you have any related conditions or symptoms before your infection that may have been related to this (if yes, what and how long)?

________________________________________________________________

| 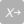 | 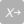 |
| --- | --- |

arthsymp Since your initial symptoms resolved (they may not have), have you had any new **joint or arthritis pain** following your infection?

- Yes (1)
- No (0)
- Don't Know (9)
- Prefer Not to Answer (99)

Display This Question:

If arthsymp = 1

arthnat Can you describe the nature of your problems? *(i.e. right knee, left elbow, etc - ask location and side of body)*

________________________________________________________________

Display This Question:

If arthsymp = 1

| 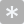 |
| --- |

arthdur How long did the symptoms last? (Days)

________________________________________________________________

Display This Question:

If arthsymp = 1

arthcurr Did you have any related conditions or symptoms before your infection that may have been related to this (if yes, what and how long)?

________________________________________________________________

| 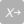 | 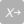 |
| --- | --- |

neusymp Since your initial symptoms resolved (they may not have), have you had any new **neurological symptoms** (muscle weakness, loss of tendon reflexes ) following your infection?

- Yes (1)
- No (0)
- Don't Know (9)
- Prefer Not to Answer (99)

Display This Question:

If neusymp = 1

neunat Can you describe the nature of your problems?

________________________________________________________________

Display This Question:

If neusymp = 1

| 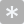 |
| --- |

neudur How long did the symptoms last? (Days)

________________________________________________________________

Display This Question:

If neusymp = 1

neucurr Did you have any related conditions or symptoms before your infection that may have been related to this (if yes, what and how long)?

________________________________________________________________

| 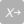 | 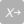 |
| --- | --- |

othrsymp Since your initial symptoms resolved (they may not have), have you noticed any new conditions or symptoms that have started since your infection?  For example, some people have reported hair loss on very rare occasions.

- Yes (1)
- No (0)
- Don't Know (9)
- Prefer Not to Answer (99)

Display This Question:

If othrsymp = 1

othrnat Can you describe the nature of your problems?

________________________________________________________________

Display This Question:

If othrsymp = 1

| 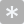 |
| --- |

othrdur How long did the symptoms last? (Days)

________________________________________________________________

Display This Question:

If othrsymp = 1

othrcurr Did you have any related conditions or symptoms before your infection that may have been related to this (if yes, what and how long)?

________________________________________________________________

text3 Thank you.  That is all the information we are asking about your health conditions.  If you are concerned about any of your symptoms, we suggest speaking to your healthcare provider.

| 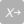 |
| --- |

incentive We have one final question.   If you were provided a monetary incentive (usually a $40 gift card), would you be willing to provide investigators a stool sample? 

 We are not collecting stool samples at this time.  We are collecting information to conduct a larger study hopefully sometime next year.  For this study we plan to collect stool samples from patients to determine how the bacteria in their bodies may have changed after their infection.  We are just asking now to learn if people would be willing to provide a sample and if not, what incentives may be needed.

- Yes (1)
- No (0)

| 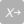 | 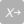 |
| --- | --- |

changeinc We are also investigating a new method of collecting samples where you are provided a wipe similar to a baby wipe that is used rather than the traditional "cup" method.  Would using this method make you more or less likely to participate or would it not matter either way?

- Yes, more likely to participate (1)
- Either way (I love to share my poop for science!) (2)
- No to either method...I don't want to know what's in my poop anymore (text box - any other incentives that would change their minds?) (0) ________________________________________________

int_comm Interviewer Comments

________________________________________________________________

text4 Thank you very much for taking the time to answer our questions today. We really appreciate your involvement in the study. Thank you and have a great day.

End of Block: Default Question Block
